# Enviromic-based kernels may optimize resource allocation with multi-trait multi-environment genomic prediction for tropical Maize

**DOI:** 10.1186/s12870-022-03975-1

**Published:** 2023-01-05

**Authors:** Raysa Gevartosky, Humberto Fanelli Carvalho, Germano Costa-Neto, Osval A. Montesinos-López, José Crossa, Roberto Fritsche-Neto

**Affiliations:** 1 Department of Genetics, Luiz de Queiroz College of Agriculture, University of São Paulo, Piracicaba, São Paulo, Brazil; 2 Centro de Biotecnología y Genómica de Plantas (CBGP, UPM-INIA), Universidad Politécnica de Madrid (UPM), Madrid, Spain; 3 Institute for Genomics Diversity, Cornell University, Ithaca, NY USA; 4 Facultad de Telemática, Universidad de Colima, Colima, Mexico; 5 International Maize and Wheat Improvement Center (CIMMYT), Km 45, Carretera Mexico-Veracruz, CP 52640 Texcoco, Edo. de México Mexico; 6 Colegio de Postgraduados, CP 56230 Montecillos, Edo. de México Mexico

**Keywords:** Genomic prediction, Training population, Enviromics, Response to selection

## Abstract

**Background:**

Success in any genomic prediction platform is directly dependent on establishing a representative training set. This is a complex task, even in single-trait single-environment conditions and tends to be even more intricated wherein additional information from envirotyping and correlated traits are considered. Here, we aimed to design optimized training sets focused on genomic prediction, considering multi-trait multi-environment trials, and how those methods may increase accuracy reducing phenotyping costs. For that, we considered single-trait multi-environment trials and multi-trait multi-environment trials for three traits: grain yield, plant height, and ear height, two datasets, and two cross-validation schemes. Next, two strategies for designing optimized training sets were conceived, first considering only the genomic by environment by trait interaction (GET), while a second including large-scale environmental data (W, enviromics) as genomic by enviromic by trait interaction (GWT). The effective number of individuals (genotypes × environments × traits) was assumed as those that represent at least 98% of each kernel (GET or GWT) variation, in which those individuals were then selected by a genetic algorithm based on prediction error variance criteria to compose an optimized training set for genomic prediction purposes.

**Results:**

The combined use of genomic and enviromic data efficiently designs optimized training sets for genomic prediction, improving the response to selection per dollar invested by up to 145% when compared to the model without enviromic data, and even more when compared to cross validation scheme with 70% of training set or pure phenotypic selection. Prediction models that include G × E or enviromic data + G × E yielded better prediction ability.

**Conclusions:**

Our findings indicate that a genomic by enviromic by trait interaction kernel associated with genetic algorithms is efficient and can be proposed as a promising approach to designing optimized training sets for genomic prediction when the variance-covariance matrix of traits is available. Additionally, great improvements in the genetic gains per dollar invested were observed, suggesting that a good allocation of resources can be deployed by using the proposed approach.

## Background

As one of the main cross-pollinated species cultivated worldwide, maize (*Zea mays* L.) has become one of the essential crops for the economic and food security of many countries [11]. Currently, maize breeding programs focus on developing single-cross cultivars, also known as hybrids. This is a consequence of the need to explore the expression of heterosis, first described by Shull [50], which has been reported to also be related to the adaptability and stability in the farm fields.

Every year, breeding programs develop thousands of hybrids to identify potential candidates with high yield and good agronomic characteristics to be released. There is however a need to evaluate the performance of those hybrids in field experiments over several years in different locations, testing a variety of traits. Nonetheless, resources are limited, and evaluations are costly and labor-intensive, making phenotyping a major bottleneck for crop improvement [16]. Therefore, to enhance the efficiency of breeding programs the use of modern breeding strategies such as phenomics, enviromics, and genomics have been used to support both predictive and analytics steps of the selection [15]. Notably genomic selection (GS) and prediction (GP) are widely used and accepted in many breeding platforms worldwide [16]. Hence, the adoption of GP models that can predict the performance early in the breeding pipeline, is of great interest to the sector [49, 57].

The use of GP emerged as a promising technique for increasing genetic gain per unit of time and reducing costs [36]. It has been extensively studied for different crops like maize, wheat, coffee, and brachiaria [9, 16, 34]. For example, it has been used to predict the performance of lines and double haploids during the initial stages of their development [26, 57]; to predict end-use quality traits [19, 27]; resistance to diseases [48]; and yield performance of maize single-crosses [5, 6, 33].

Among the factors that can impact GP accuracy, the establishment of a good training set (TRS) is key [18, 43]. In addition to high-quality genotypic and phenotypic data, it is important that the TRS is representative in terms of population size, diversity, and that it is genetically related to new breeding candidates in the testing set (TS) it is expected to predict [4, 16, 19, 22]. However, complexities due to differences in allele frequency and linkage disequilibrium patterns between the genotypes in the TRS and the TS may limit the prediction accuracy of the TRS. Thus, the question is whether all available data or a subset of it should be used when optimizing the GP models [30].

As phenotyping is a high-cost activity, the adoption of strategies for optimizing the TRS could represent important gains in breeding efficiency, such as reduction of phenotyping costs, efficient resource allocation, and the possibility to increase the prediction ability (PA) of unobserved genotypes [3, 20, 21, 27, 43, 45, 51] and unobserved environments [12]. Despite the recent advances in this field, there is a lack of knowledge about what is the optimal distribution of genotypes in multi-environment trials to achieve the best balance between the number of genotypes tested in the field and the prediction ability of GS models, thus maximizing the selection gain under a fixed budget [24]. Additionally, in multi-trait multi-environment trials (MTMET), we could ask which traits should be evaluated in each genotype × environment combination.

Hence, the ideal strategy in GS is to design optimized training sets for GP, which maintains the accuracy of prediction at satisfactory levels using a training set that is representative [12, 18]. In this context, many studies have been conducted to establish the balance between investment and efficiency through different methods, experimental design, statistical analysis, and TRS composition [21]. For instance, the genetic algorithm to design training populations developed by Akdemir [2] was tested by Pinho Morais et al. [43] for several population sizes. In addition, the responses were compared with randomly selected small training set populations, concluding that optimizing TRS can be effective in obtaining satisfactory accuracies.

Using multi-trait (MT) models, Lado et al. [27] tested other resource allocation strategies by comparing the prediction accuracy of different levels of availability of phenotypic information for the target trait. Accordingly, Lado et al. [27] tested different TRS sizes ranging from 80 to 10%, then included the phenotypic data of all individuals for correlated traits, and finally, considered balanced and unbalanced scenarios. In the balanced scenarios, phenotypic data were provided for all correlated traits used in the model. In contrast, in the unbalanced scenarios, phenotypic data were not provided for all traits, but rather for one, across both the TRS and TS sets. The results showed no loss in PA when reducing TRS for a target trait by up to 30% when using full information on correlated traits. Additionally, the unbalanced phenotyping approach for correlated traits performed better than the balanced one to optimize the TRS.

Costa-Neto et al. [12] investigated the inclusion of dominance effects and envirotyping data into a single-trait multi-environment trial (STMET) scenario. Especially for traits with low heritability and highly influenced by the environment, the environmental covariates (ECs) can increase PA for new environments or newly developed hybrids by tracking variation sources, and environment resources and reducing the error variance. Taking advantage of some new tools, such as the EnvRtype R package [13], it is possible to compose a covariance matrix among trials, making it possible to dissect the G × E interaction and build environmental relationship matrices for GP, which will better explain the sources of non-genetic variation. Additionally, the envirotyping information can be associated with genomic data in genetic algorithms to better select genotypes and target environments that are more informative in terms of G × E [12].

Lopez-Cruz, and de los Campos, G [30]. proposed a prediction method (sparse selection index, SSI) that identifies a customized training set for every individual in the prediction set. The SSI integrates a sparsity-inducing penalty into the selection index methodology [31], which leads to sparse selection indices. Results from Lopez-Cruz, and de los Campos, G [30]. in two wheat datasets, and Lopez-Cruz et al. [28] in several multi-year maize datasets, showed that the SSI outperformed the genomic-BLUP (GBLUP [53];) in terms of PA. However, the SSI methods for optimizing the TRS do not incorporate multi-trait and/or multi-environment (or multi-year) structures.

Other studies proposed different methods of optimizing training sets for GS. Rincent et al. [46] used prediction error variance (PEVmean) and coefficient of determination (CDmean) to select individuals to constitute TRS; Isidro et al. [20] used stratified sampling and stratified CD; Akdemir & Isidro-Sánchez [3] studied two different scenarios for optimization, “untargeted” and “target”, integrated to the concept of Design of Experiments (DOE). A recent review from Isidro y Sánchez and Akdemir [21] put together different training set optimization studies and perspectives.

Based on the above considerations, the main objective of this study was to test the performance of optimized training sets (OTS) for MTMET, along with the use of environmental covariates matrices (W matrix) in genomic prediction models. Thus, we aimed to diminish the phenotypic labor due to lower but optimally selected population sizes while keeping the prediction ability at satisfactory levels. Finally, we were interested in comparing the response to selection of OTS with MTMET and phenotypic selection, to estimate the genetic gain per unit of dollar invested. For that, we (i) fitted and compared the performance of five different prediction models, the baseline GBLUP with additive + dominance effects (M1), then added G × E (M2), including enviromic data (W) (M3), then fitted W + G × E (M4), and finally achieving W + G × W (M5); (ii) estimated the genomic prediction ability of the five prediction models for single-trait multi-environment trials (STMET) and MTMET for three traits in two tropical maize datasets; and (iii) estimated the genomic prediction ability using OTS with controlled unbalancing of G, E and trait (T) information, selected by a combination of two genetic algorithms, the APY [38, 39] and the LA-GA-T from the STPGA R package [2].

## Results

### Descriptive statistics

Pearson’s correlation between traits was calculated for each dataset using the BLUEs obtained in the first step. As a result of the variable transformation, EH and PH assumed a negative correlation with GY, which agrees with the selection targets. Therefore, we attempted to increase GY while fixing or reducing EH and PH. For the *HEL dataset*, GY had a moderately negative correlation with PH and EH, of − 0.55 and − 0.58, respectively, while PH and EH had a high positive correlation of 0.82. For the *USP dataset*, GY had weak negative correlations with PH and EH, of − 0.44 and − 0.33, respectively, while PH and EH had a high positive correlation of 0.70.

Estimated heritability was intermediate to high: for the *HEL dataset*, trait heritability was 0.62, 0.78, and 0.80 for GY, PH, and EH, respectively; for the *USP dataset*, heritability was 0.56 for GY, 0.84 for PH, and 0.89 for EH.

As expected, a lower correlation was found between the complex trait GY and the other traits than for PH and EH. Moreover, GY had a lower heritability than EH and PH (see Table 1).

**Table 1:** Phenotypic correlation (r_(x,y)_) between traits and broad-sense heritability (H^2^) for HEL and USP datasets

Dataset	Trait	r_**(x,y)**_			H^**2**^
		GY	PH	EH	
**HEL**	GY	–	−0.55	−0.58	0.62
	PH	–	–	0.82	0.78
	EH	–	–	–	0.80
**USP**	GY	–	−0.44	−0.33	0.56
	PH	–	–	0.70	0.84
	EH	–	–	–	0.89

### Optimized Training Sets (OTS)

The first result of selecting information to form the training sets, using the APY algorithm, returned the sample sizes for each kernel, as described in Table 2 below.

**Table 2:** Sample sizes output from the APY algorithm. Sample sizes and respective percentages (%) were obtained and calculated for each dataset (HEL and USP), kernel (GET and GWT), and optimized training set scenario (OTS 1, OTS 2, and OTS 3). GET: genotype × environment × trait; GWT: genotype × environmental covariates × trait. Percentages were obtained based on dataset size

	GET			GWT		
	OTS 1	OTS 2	OTS 3	OTS 1	OTS 2	OTS 3
**HEL**						
Sample size	155	297	427	102	197	291
%	**7**	**13.4**	**19.2**	**4.6**	**8.9**	**13.1**
**USP**						
Sample size	267	521	763	107	212	314
%	**3.6**	**7**	**10.2**	**1.4**	**2.8**	**4.2**

The sample size differs depending on the kernel and germplasm. Notice that regardless of the germplasm, sample sizes are larger for GET than for GWT (155 vs. 102 for *HEL*; and 267 vs. 107 for *USP*), suggesting so far that the latter has greater optimization capability.

To minimize the stochastic error, we took three samples from the LA-GA-T algorithm. Thus, the first training set scenario, OTS 1, has three random samples. In the second scenario, OTS 2, we made the three possible pair combinations between the three samples, resulting again in three different samples, about twice the size of OTS 1. Finally, we joined the three samples from OTS 1 to build a larger optimized training set, OTS 3.

**Fig. 1:**
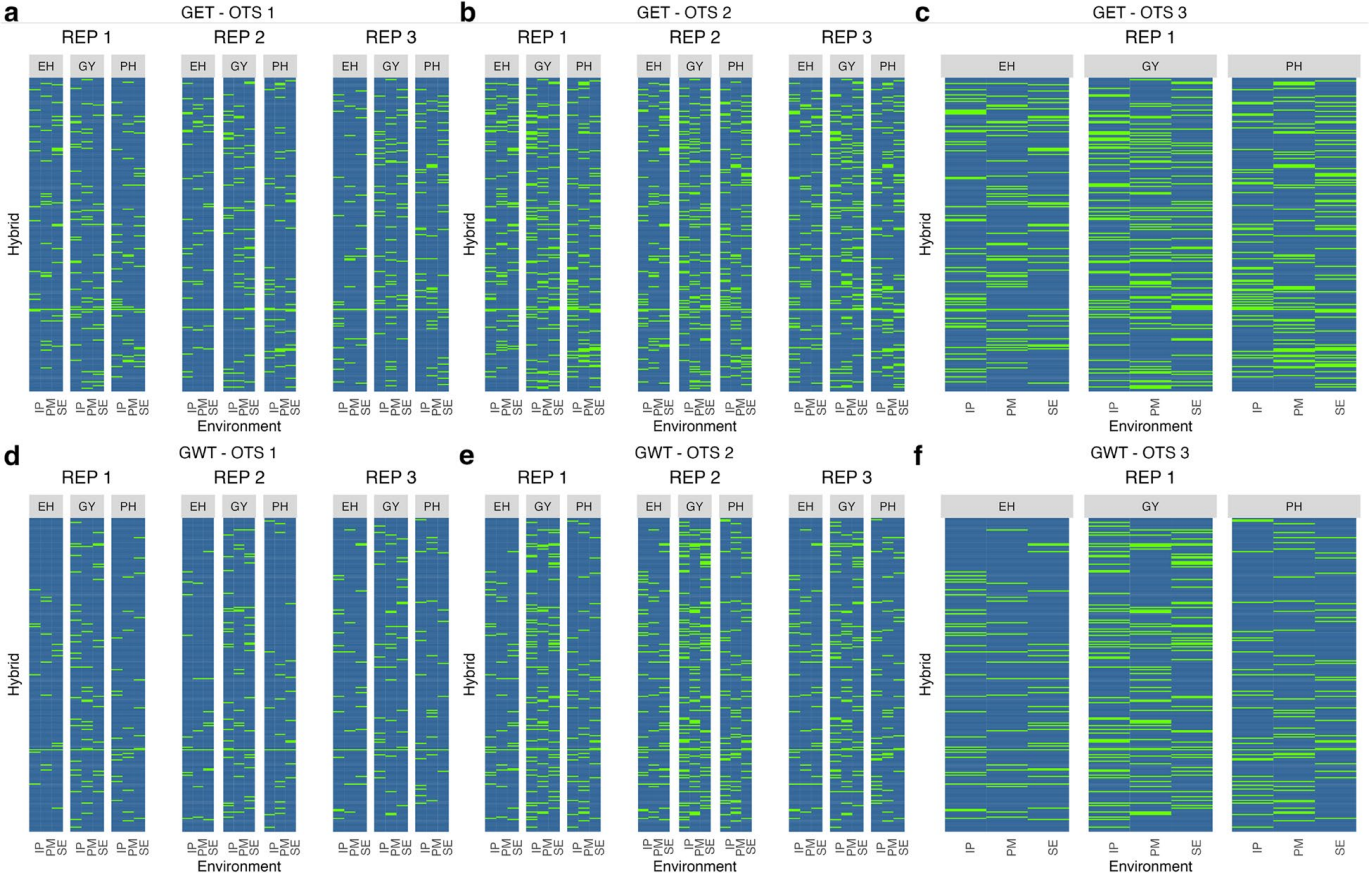
Heatmap of OTS (optimized training sets) graph for the Helix dataset. **a** OTS 1 for kernel GET. **b** OTS 2 for kernel GET. **c** OTS 3 for kernel GET. **d** OTS 1 for kernel GWT. **e** OTS 2 for kernel GWT. (f) OTS 3 for kernel GWT. In green (green = 1 or presence of this information in the training set) we see the distribution of the information selected to form the training population, for each trait x environment and replication inside kernels, while in blue (blue = 0 or absence of the information in the TRS), we see what formed our testing set, or what was predicted. The solid line that crosses all the graphs represents the genotype used as a check. The environments on the x-axis: IP (Ipiaçu), PM (Patos de Minas), and SE (Sertanópolis); the traits under study: EH (ear height), GY (grain yield), and PH (plant height). The kernels: GET (genotype ***×*** environment ***×*** trait) and GWT (genotype ***×*** environmental covariates ***×*** trait) were used as the base to select the information

**Fig. 2:**
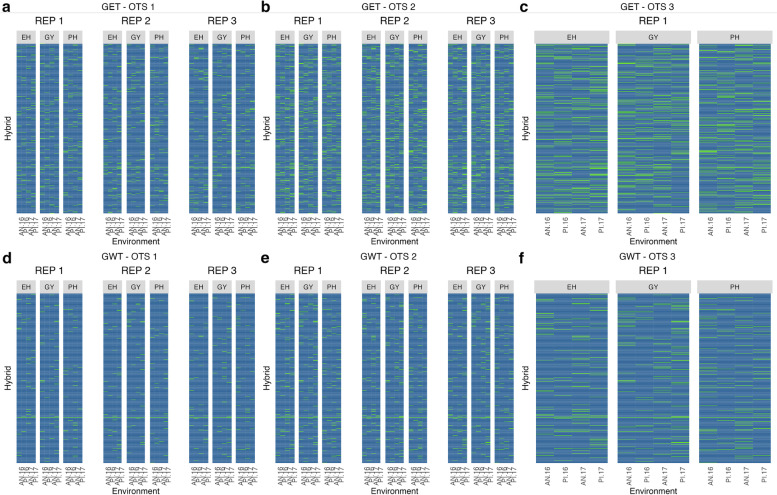
Heatmap of OTS (optimized training sets) graph for the USP dataset. **a** OTS 1 for kernel GET. **b** OTS 2 for kernel GET. **c** OTS 3 for kernel GET. **d** OTS 1 for kernel GWT. **e** OTS 2 for kernel GWT. **f** OTS 3 for kernel GWT. In green (green = 1 or presence of this information in the training set) we see the distribution of the information selected to form the training population, for each trait x environment and replication inside kernels, while in blue (blue = 0 or absence of the information in the TRS), we see what formed our testing set, or what remained to be predicted. The solid line that crosses all the graphs represents the genotype used as a check. The environments on the x-axis: AN.16 (Anhembi 2016), PI.16 (Piracicaba 2016), AN.17 (Anhembi 2017), and PI.17 (Piracicaba, 2017); the traits under study: EH (ear height), GY (grain yield) and PH (plant height). The kernels: GET (genotype × environment × trait) and GWT (genotype × environmental covariates × trait) are used as the base to select the information

Individuals were randomly sampled (with replacement) by combining information of genotype × environment × trait. For that reason, samples were trait and environment specific. The difference in selected information among the three samples from the different OTS scenarios can be seen in the heatmaps for HEL (Fig. 1a-f) and USP (Fig. 2a-f).

### Prediction abilities of the five models over STMET and MTMET scenarios

As mentioned in the material and methods section, we assessed PA in two ways, however, general results will be presented referring to the average of environments across traits, while specific cases from the PA per trait per environment results will be highlighted.

### HEL dataset

#### Single-trait multi-environment trial analysis

CV1 resulted in PAs varying from 0.53 to 0.73 (Table 3). PAs for GY varied by up to 20% between models, while for EH and PH, differences were minimal, ranging from 0 to 2%. However, the results per environment, Sertanopolis (SE) showed the lowest PA for GY, mainly when models without the G × E interaction (**M1** and **M3**) were used. In contrast, models including G × E (**M2** and **M4**) increased PA by 100 to 104%, and G × W (M5) increased PA by 92% for this particular trait × environment combination. CV2 (Table 3) produced the same patterns as those for CV1, with PAs ranging from 0.52 to 0.79. Similarly, for GY at SE, PA benefited with increases between 61 to 68% when models with G × W (**M5**) and G × E interactions (**M2** and **M4**) were used, respectively.

**Table 3:** Prediction ability of the HEL dataset for single trait multi-environment trial (STMET) analysis for five models: EAD (M1); EAD + GE (M2); EADW (M3); EADW + GE (M4) and EADW + GW (M5), for both cross-validations CV1 and CV2. PAs were calculated by environment for grain yield (GY), ear height (EH) and plant height (PH). *μ* is the average PA across environments and % is the difference between that PA and the highest PA. Standard deviation appears before the “±” sign

Trait		M1	M2	M3	M4	M5
CV1						
GY	***μ***	0.53 ± 0.20	**0.66 ± 0.13**	0.53 ± 0.20	**0.66 ± 0.13**	0.64 ± 0.15
	**%**	−20	–	−20	–	−3
EH	***μ***	0.69 ± 0.06	**0.70 ± 0.06**	0.69 ± 0.06	**0.70 ± 0.06**	**0.70 ± 0.06**
	**%**	−1	–	−1	**–**	–
PH	***μ***	0.72 ± 0.06	**0.73 ± 0.06**	0.72 ± 0.06	**0.73 ± 0.06**	**0.73 ± 0.06**
	**%**	−2	–	−2	–	–
CV2						
GY	***μ***	0.52 ± 0.17	**0.68 ± 0.12**	0.52 ± 0.18	**0.68 ± 0.12**	0.67 ± 0.13
	**%**	−24	–	−24	–	−2
EH	***μ***	**0.75 ± 0.06**	**0.75 ± 0.05**	**0.75 ± 0.05**	**0.75 ± 0.05**	**0.75 ± 0.05**
	**%**	–	–	–	**–**	–
PH	***μ***	0.78 ± 0.05	**0.79 ± 0.05**	0.78 ± 0.05	**0.79 ± 0.05**	**0.79 ± 0.05**
	**%**	− 2	–	−2	–	–

Comparing CV1 and CV2, PAs increased on average by 3 to 8%. In general, **M4** showed the best performance. Overall, the greatest difference was observed for GY, between models **M1** and **M3** (without G × E interaction), and **M2** and **M4 (**with G × E interaction), where **M4** and **M2** outperformed **M3** and **M1** by a range of 25 and 30%; however, **M5** also outperformed **M3** and **M1** by 29%.

#### Multi-trait multi-environment trial analysis

As for the STMET analysis, the results of the multi-trait (MTMET) analysis showed a similar pattern of responses, both for CV1 and CV2, varying from 0.54 to 0.73 and 0.53 to 0.79, respectively (Table 4). Nevertheless, we noticed that PAs increased between 61 and 68% for CV1 at SE, when exploring the G × W and G × E interaction effects. The PA variation for GY between models was very similar to that obtained for STMET (0-24%). For EH and PH, accuracy variation ranged from 0 to 1%. In general, **M4** performed better.

**Table 4:** Prediction ability of the HEL dataset for multi-trait multi-environment trial (MTMET) analysis for the five models under study: EAD (M1); EAD + GE (M2); EADW (M3); EADW + GE (M4) and EADW + GW (M5), for both cross-validation schemes CV1 and CV2. PAs were calculated per environment for grain yield (GY), ear height (EH), and plant height (PH). *μ* is the average PA across environments and % is the difference between that PA and the highest PA. Standard deviation appears before the “±” sign

Trait		M1	M2	M3	M4	M5
**CV1**						
GY	***μ***	0.54 ± 0.18	0.66 ± 0.12	0.54 ± 0.17	**0.67 ± 0.12**	0.65 ± 0.14
	**%**	− 19	–	− 19	–	− 3
EH	***μ***	0.70 ± 0.06	0.70 ± 0.06	0.70 ± 0.06	**0.71 ± 0.06**	0.70 ± 0.06
	**%**	− 1	− 1	− 1	**–**	− 1
PH	***μ***	0.72 ± 0.06	**0.73 ± 0.06**	0.72 ± 0.06	**0.73 ± 0.06**	**0.73 ± 0.06**
	**%**	− 1	–	− 1	–	–
**CV2**						
GY	***μ***	0.53 ± 0.16	**0.68 ± 0.11**	0.53 ± 0.16	**0.68 ± 0.11**	0.67 ± 0.12
	**%**	− 22	–	− 22	–	− 1
EH	***μ***	0.70 ± 0.06	**0.75 ± 0.06**	**0.75 ± 0.06**	**0.75 ± 0.06**	**0.75 ± 0.06**
	**%**	–	–	–	**–**	–
PH	***μ***	0.78 ± 0.05	**0.79 ± 0.05**	0.78 ± 0.05	**0.79 ± 0.05**	**0.79 ± 0.05**
	**%**	− 1	–	− 1	–	–

Comparing CV1 and CV2, the PAs increased between 2.4 to 7.7%, with higher differences for GY. While comparing STMET and MTMET, PAs rose from 0 to 1.4%, where the differences were higher for GY and nonexistent for PH.

### USP dataset

#### Single-trait multi-environment trial analysis

CV1 showed PAs varying from 0.46 to 0.65 (Table 5). The isolated environment Piracicaba *x* 2017 (PI.17) presented an inferior performance for EH when models without G × E interaction (**M1** and **M3**) were used. In contrast, models with G × E (**M2** and **M4**) increased the accuracy up to 440% for EH at PI.17, and models with G × W (**M5**) increased PA by 420%. When considering the average between environments, PA for EH only varied from 0 to 7% between models. CV2 produced similar patterns as those for CV1. For EH in the PI.17 environment, PA increased by 340% when models with G × E interactions were used and by 320% when using G × W. In general, **M2** and **M4** showed the best performances.

**Table 5:** Prediction ability of the USP dataset for single trait multi-environment trial (STMET) analysis for the five models under study: EAD (M1); EAD + GE (M2); EADW (M3); EADW + GE (M4) and EADW + GW (M5), for both cross-validation schemes CV1 and CV2. PAs were calculated by environment for grain yield (GY), ear height (EH), and plant height (PH). ***μ*** is the average PA across environments and % is the difference between that PA and the highest PA. Standard deviation appears before the “±” sign

Trait		M1	M2	M3	M4	M5
**CV1**						
GY	***μ***	0.46 ± 0.06	**0.48 ± 0.05**	0.46 ± 0.06	**0.48 ± 0.05**	0.47 ± 0.05
	**%**	−4	–	−4	–	−1
EH	***μ***	0.57 ± 0.30	**0.62 ± 0.22**	0.57 ± 0.30	**0.62 ± 0.22**	**0.62 ± 0.22**
	**%**	−8	**–**	−8	**–**	–
PH	***μ***	**0.65 ± 0.05**	**0.65 ± 0.05**	**0.65 ± 0.05**	**0.65 ± 0.05**	**0.65 ± 0.05**
	**%**	–	–	–	**–**	**–**
**CV2**						
GY	***μ***	0.48 ± 0.05	**0.51 ± 0.05**	0.48 ± 0.05	**0.51 ± 0.05**	0.50 ± 0.05
	**%**	−6	**–**	−6	**–**	−2
EH	***μ***	0.58 ± 0.31	**0.61 ± 0.24**	0.58 ± 0.31	**0.61 ± 0.24**	**0.61 ± 0.25**
	**%**	−5	**–**	−5	**–**	−1
PH	***μ***	**0.70 ± 0.04**	**0.70 ± 0.04**	**0.70 ± 0.04**	**0.70 ± 0.04**	**0.70 ± 0.04**
	**%**	–	–	–	–	**–**

#### Multi-trait multi-environment trial analysis

As for the STMET analysis, the results of the MTMET analysis showed similar response patterns, both for CV1 and CV2 (Table 6). Nevertheless, PAs increased between 4 and 8%. As for STMET, at PI.17 we obtained low PA for EH in MTMET, but by exploring G × E, accuracy increased up to 333%.

**Table 6:** Prediction ability of the USP dataset for multi-trait multi-environment trials (MTMET) analysis for the five models under study: EAD (M1); EAD + GE (M2); EADW (M3); EADW + GE (M4) and EADW + GW (M5), for both cross-validation schemes CV1 and CV2. PAs were calculated by environment for grain yield (GY), ear height (EH), and plant height (PH). ***μ*** is the average PA across environments and % is the difference between that PA and the highest PA. Standard deviation appears before the “±” sign

Trait		M1	M2	M3	M4	M5
**CV1**						
GY	***μ***	0.46 ± 0.06	0.47 ± 0.05	0.46 ± 0.06	**0.48 ± 0.05**	0.47 ± 0.05
	**%**	−4	–	−4	**–**	−2
EH	***μ***	0.57 ± 0.30	**0.62 ± 0.22**	0.57 ± 0.30	**0.62 ± 0.22**	**0.62 ± 0.22**
	**%**	−8	–	−8	**–**	–
PH	***μ***	**0.65 ± 0.05**	**0.65 ± 0.05**	**0.65 ± 0.05**	**0.65 ± 0.05**	**0.65 ± 0.05**
	**%**	–	–	–	–	**–**
**CV2**						
GY	***μ***	0.48 ± 0.05	**0.51 ± 0.05**	0.48 ± 0.05	**0.51 ± 0.05**	**0.51 ± 0.05**
	**%**	−6	–	−6	**–**	–
EH	***μ***	0.58 ± 0.31	**0.61 ± 0.25**	0.58 ± 0.31	**0.61 ± 0.25**	**0.61 ± 0.25**
	**%**	−5	–	−5	**–**	–
PH	***μ***	**0.70 ± 0.04**	**0.70 ± 0.04**	**0.70 ± 0.04**	**0.70 ± 0.04**	**0.70 ± 0.04**
	**%**	–	–	–	–	**–**

Overall, M4 performed better for all scenarios in this section. We also noticed that the inclusion of interaction terms in the models, regardless of whether it was G × E or G × W, always increased PA.

### The prediction ability for OTS scenarios

Like the two previous scenarios (STMET and MTMET) for the optimized training sets (OTS), the five models M1 to M5 were also tested. However, the model with the best performance was the **M4**, so only this result will be presented and discussed from hereon. Remember that M4 is the model that includes G × E and W.

**Fig. 3:**
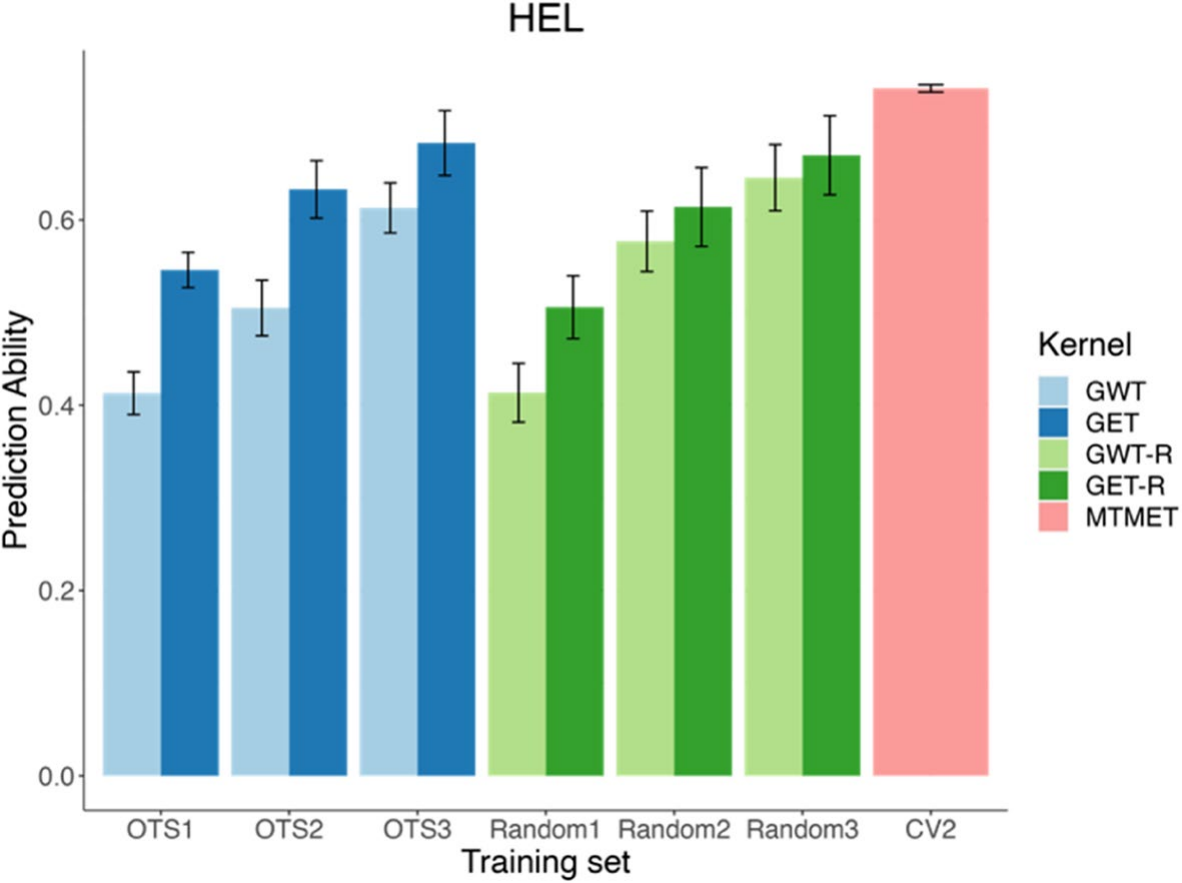
The bar plot shows the prediction ability (average of environments and traits) according to different training sets (TRS) used for genomic prediction for the HEL (Helix) dataset. The plot represents the training sets by OTS1, OTS2, OTS3, Random1, Random2, Random3 and CV2. OTSs are our optimized training sets, that increase in size (from 4.6 to 19.2% of TRS) by combining one, two, or three replications of the populations selected by the LA-GA-T algorithm. Random’s are our randomly sampled training sets with the same size of OTSs. CV2 is our benchmark scenario, where we used 70% of TRS, and a cross-validation scheme (CV2), under a multi-trait-multi environment model (MTMET). GET (genotype x environment x trait) and GWT (genotype *x* environmental covariates *x* trait) are the kernels used as the basis for the selection of information by the LA-GA-T algorithm. GET-R and GWT-R represents the equivalent of OTS, in terms of sample size, but for the random samples. Error bars represents the standard error

*HEL dataset:* On average, with OTS 1, we achieved PAs across traits of 0.55 and 0.41 for GET and GWT kernels, respectively (Table 7). These values increased to 0.63 (+ 14.5%) and 0.51 (+ 24.4%), from OTS 1 to OTS 2, then to 0.68 (+ 7.9%) and 0.61 (+ 21.6%), from OTS 2 to OTS 3. The PA benchmark, obtained through MTMET-CV2 (our reference value), was 0.74 (Table 7 and Fig. 3), this value is an average between the traits’ PA. Figure 3 also shows that the PA of optimized training samples are better than random samples of the same size for small training sets, by up to 7.9% when comparing Random1 with OTS1. The differences in PA will, however, decrease with increasing sample size, and Random samples may outperform optimized samples in some cases.

**Table 7:** Predictive abilities, per trait (EH, GY, and PH) and average across traits (*μ*), as training set size increases (OTS 1, 2, and 3), for GET (genotype x environment x trait) and GWT (genotype x environmental covariates x trait) kernels, for the HEL (Helix) and USP (University of Sao Paulo) datasets. Ne: number of information used as the training set; prediction accuracies for EH: ear height, GY: grain yield, and PH: plant height; %: the percentage increase between that value and the value immediately above; OTS: optimized training set. Here, Ne was added with the check’s information into the original sample sizes, being 9 information for HEL and 12 information for USP

		PREDICTION ABILITY									
				GET					GWT		
	OTS	Ne	EH	GY	PH	***μ***	Ne	EH	GY	PH	***μ***
**HEL**	1	**155**	0.61	0.47	0.56	0.55	**102**	0.43	0.42	0.38	0.41
	2	**306**	0.67	0.56	0.66	0.63	**206**	0.54	0.50	0.48	0.51
	%	+ 97	+ 9.8	+ 19.1	+ 17.9	+ 14.5	+ 100	+ 25.6	+ 19	+ 26,3	+ 24.4
	3	**436**	0.72	0.60	0.72	0.68	**300**	0.64	0.60	0.63	0.62
	%	+ 42	+ 7.5	+ 7,1	+ 9	+ 7.9	+ 45	+ 18,5	+ 20	+ 31.2	+ 21.6
**USP**	1	**267**	0.47	0.28	0.48	0.41	**107**	0.22	0.19	0.32	0.24
	2	**533**	0.52	0.34	0.56	0.47	**224**	0.31	0.23	0.36	0.30
	%	+ 99.6	+ 10.6	+ 21.4	+ 16.7	+ 14.6	+ 87.5	+ 40.9	+ 21	+ 12.5	+ 25
	3	**775**	0.56	0.40	0.61	0.52	**326**	0.43	0.35	0.54	0.44
	%	+ 45.4	+ 7.7	+ 17.6	+ 8.9	+ 10.6	+ 43	+ 38.7	+ 52.2	+ 50	+ 46.7

**Fig. 4:**
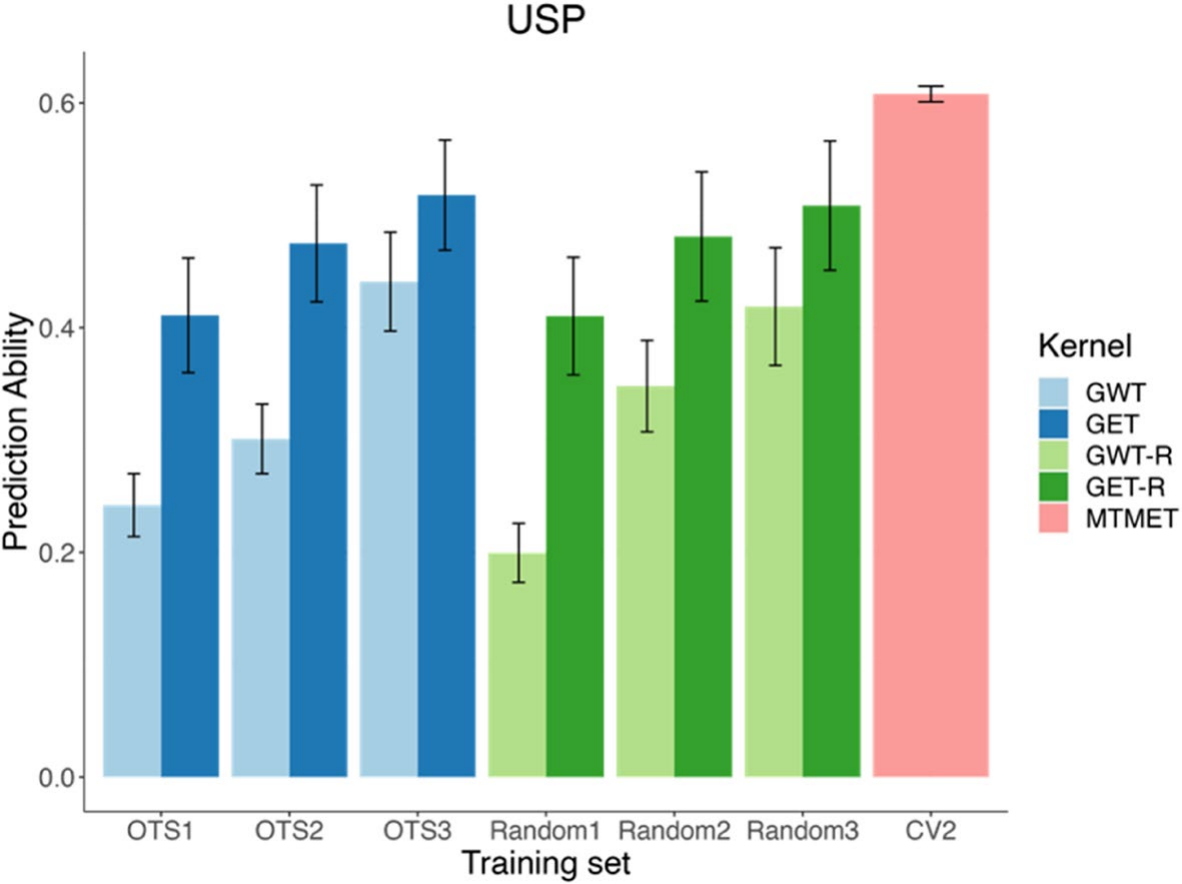
The bar plot shows the prediction ability (average of environments and traits) according to different training sets (TRS) used for genomic prediction for the USP (University of Sao Paulo) dataset. The plot represents the training sets by OTS1, OTS2, OTS3, Random1, Random2, Random3, and CV2. OTSs are our optimized training sets, that increase in size (from 1.4 to 10.2% of TRS) by combining one, two, or three replications of the populations selected by the LA-GA-T algorithm. Random’s are our randomly sampled training sets with the same size of OTSs. CV2 is our benchmark scenario, where we used 70% of TRS, and a cross-validation scheme (CV2), under a multi-trait-multi environment model (MTMET). GET (genotype *x* environment *x* trait) and GWT (genotype *x* environmental covariates *x* trait) are the kernels used as the basis for the selection of information by the LA-GA-T algorithm. GET-R and GWT-R represents the equivalent of OTS, in terms of sample size, but for the random samples. Error bars represents the standard error

*USP dataset:* the average PA across traits for OTS 1 was 0.41 and 0.24 for GET and GWT kernels, respectively. Following the same pattern as the HEL dataset, when we increased the size of TRS, those values increased to 0.47 (+ 14.6%) and 0.30 (+ 25%) for OTS 2, to 0.52 (+ 10.6%) and 0.44 (+ 46.7%) for OTS 3, while the maximum PA obtained by the benchmark was 0.61 (Table 7 and Fig. 4). As observed for the HEL dataset, it can be noted in Fig. 4 that when the training set is smaller, OTS is better than Random, by up to 20.1% (GWT kernel – OTS1). Nonetheless, the larger the sample sizes, the smaller the differences between scenarios.

Note in Table 7 that, with similar percentage increases in training set sizes, the GWT kernel yields a much larger percentage increase in PA. This result is true for both datasets, but especially for the USP one, for which the total amount of information is much larger. It is important to remember that the number of information refers to the combinations of genotype x environment x trait.

### Response to selection per amount invested

The genetic gain per 10,000 USD invested was estimated for each dataset × OTS scenario, primarily to compare the efficiency of genomic prediction at the kernel level (GET vs. GWT). Then, the estimated genetic gain was compared between scenarios as follows: OTS vs. MTMET CV2 (benchmark) and OTS vs. phenotypic selection (PS).

**Fig. 5:**
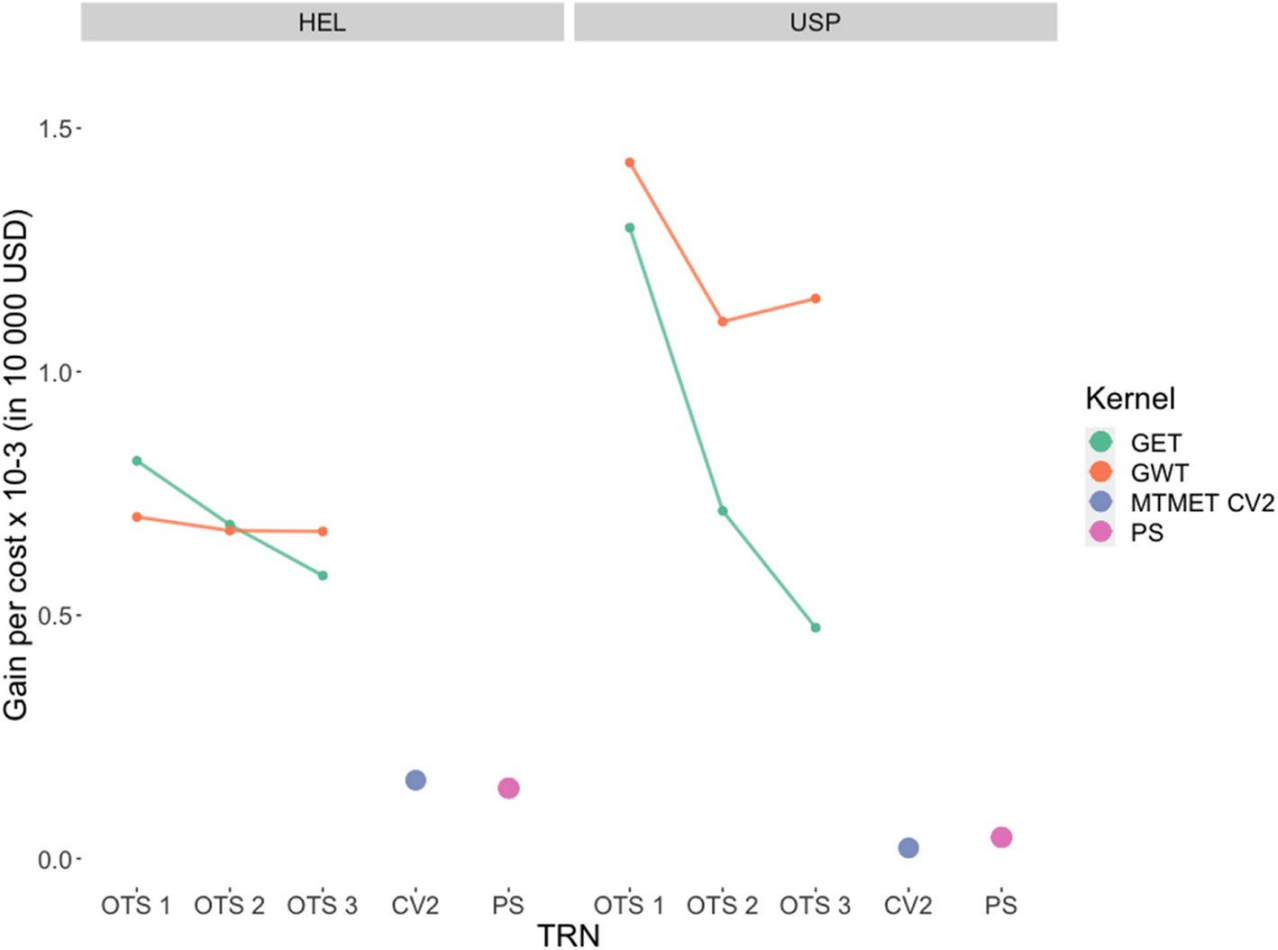
Genetic gain per cost × 10^− 3^ (per 10,000 dollars invested) across training set scenarios for the HEL and USP datasets, comparing the two optimization kernels (GET and GWT) and the benchmark (MTMET CV2, where TRS = 70%). The cost includes the phenotyping of TRS (on average 3 USD per trait per plot) and the whole dataset’s genotyping (20 USD per sample). Where GET: genotype x environment x trait; GWT: genotype x environmental covariates x trait; MTMET CV2: multi-trait multi-environment model with cross-validation CV2; PS: phenotypic selection

For the *HEL dataset*, results showed an inversion of the response to selection per amount invested between kernels across scenarios. Even though GET had a higher cost per gain for OTS1 than GWT, it decreased its response throughout the scenarios while GWT remained constant. The genetic gain per 10,000 USD invested ranged from 0.80 × 10-3 vs 0.70 × 10-3 (OTS 1), for GET and GWT respectively, and to 0.58 × 10-3 vs 0.67 × 10-3 in OTS 3. For PS, the gain was 0.14 × 10-3 (Fig. 5).

For the *USP dataset,* the genetic gain per 10,000 USD invested ranged from 1.29 × 10^− 3^ vs. 1.42 × 10^− 3^ for GET and GWT, respectively, to 0.47 × 10^− 3^ vs. 1.15 × 10^− 3^. For PS, the gain was 0.04 × 10^− 3^ (Fig. 5). Note that for this dataset, GWT outperformed GET for all OTS scenarios.

As a matter of comparison, we did the same procedure for the benchmark scenario MTMET CV2 (using **M4**), and the gains per investment were 0.16 × 10^− 3^ for HEL and 0.02 × 10^− 3^ for the USP dataset.

As presented in earlier sections, the prediction ability varies, among other factors, depending on the size of the TRS according to the tested scenarios (STMET, MTMET, and OTS). Here we present the genetic gains in each TRS scenario under a fixed budget of 10,000 dollars, enabling a direct comparison. For small training population sizes, as in OTS, predictive abilities are lower, but the investments in TRS are also lower, which results in a satisfactory gain per dollar invested.

## Discussion

The major goal of GS can be defined as increasing the genetic gain with no increase in costs compared to only phenotypic selection [16, 57], thus compensating for the loss in prediction ability with the gains in response to selection.

The traits evaluated here are moderate to strongly positively correlated by Pearson’s correlation coefficient. However, the selection targets in breeding programs are in opposite directions. While we want to increase grain yield, we want to decrease plant height and stabilize ear height. Dwarf plants with high yields are already a reality in other crops like wheat and rice. In maize, dwarfing mutant genes have been studied, and unlike wheat and rice, PH in maize is a quantitative trait that affects other plant characteristics like yield losses, making it difficult to apply [10]. Since we want all the great attributes simultaneously, we created an adjusted EH, which measures the distance from the actual phenotype to an ideal ideotype, defined here as 80 cm. Then traits assume a negative correlation between them, allowing us to select all the traits concurrently [56].

As reported by Ibba et al. [19], as well as Werner et al. [57], prediction accuracy is population-specific and depends on several other factors such as traits and environments. These factors include but are not limited to, the model chosen, the traits to be predicted, the trait heritability, the correlation between traits, the environments where trials were performed or new environments to be predicted, and their correlation. So, the results here were no exception. Furthermore, differences in prediction ability between the two datasets, which occurred for STMET, MTMET, and OTS’s kernels (**GET** and **GWT**), can be partially attributed to differences in Pearson’s correlation coefficient between traits, since the prediction ability is directly related to the correlation between traits [27].

We saw that some models outperformed others, which was also expected since they include additional terms such as the environmental covariates matrix (**W**) and interaction terms (**G** × **E** and **G** × **W**), which can better capture the portion of variance explained by the model [5]. The results of Dias et al. [17] suggested that GBLUP models that contain additivity, dominance, and G × E interaction should be preferred for predicting the performance of newly developed hybrids in any MET analyses, as was the case with STMET and MTMET under CV-1, and OTS scenarios.

The present study used two cross-validation schemes, CV-1, and CV-2 [7], to evaluate the PA for STMET and MTMET. Across all combinations of dataset × model × single or multi-trait, CV-2 outperformed CV-1 because it included phenotypic information of genotypes in some environments but not in others, which led to higher PAs. To benchmark and compare the five different prediction models, we used cross-validation Schemes CV-2 and CV-1 along with STMET and MTMET [57]. Thus, based on this prior validation, the model with the highest accuracies (**M4** - G × E + W) was chosen for the prediction using the optimized training set populations, and those validations were also used as a comparison parameter for prediction accuracies while increasing the sample sizes of TRS (see Figs. 3 and 4). Randomly selected training populations with the same size of the OTS were also compared with OTS prediction accuracy. When predicting GEBVs of the OTSs and Random scenarios, a pre-known covariance matrix of traits was used.

It should be noted that the model with the best performance includes the G × E interaction component, which coincides with what has already been reported by several authors [1, 7, 17, 24, 40, 47] and more recently, by Jarquin et al. [25]. Furthermore, adding the interaction effect G × W, as in M5, increases PA when compared to the main effects models (M1 and M3), but not as much as models containing the G × E (M2 and M4), like what Robert et al. [47] and Jarquin et al. [25] reported. However, G × W has the advantage when predicting new environments [14, 25, 47], which have not been tested here. Costa-Neto et al. [14] that including the W matrix increases PA, especially for untested hybrids and/or untested environments, by better explaining sources of variation, capturing the environment potential per se, and the interaction with the genotypes. Nevertheless, in the present study, and according to what was presented by [25], the inclusion of the W matrix alone (M3) did not improve PA, being similar to M1, and the inclusion of W with G × E in M4 gave similar results as the G × E alone (M2). Nevertheless, the W matrix can optimize complex information, as we saw in our OTS’ scenarios, and optimize trials [25]. Also, MTMET models showed a small PA increase compared with STMET, especially for models containing the G × E term [33, 35].

Studies involving SNP data, such as GP, require the inverse of the genomic relationship matrix (GRM). However, as the number of individuals to be evaluated increases, the computational cost of this matrix’s inversion is relatively high, with limitations in memory and time. To minimize this problem, Misztal [38] proposed an algorithm for proven and young animals (APY). Using the APY, it is not necessary to make a complete inversion of the GRM, since its output returns the number of individuals (n) needed to sample 98% of the population variation. Then, the inverse of GRM can be obtained by recursion, based on the information of the core individuals. However, this algorithm does not specifically indicate which genotypes we should select as core individuals. Hence, it is necessary to take a random sample of the population. Here we extended the APY to plants. Since the APY does not provide information on which genotypes should be included in the core population, another algorithm was used to select these individuals efficiently. We used the genetic optimization algorithm for selecting subpopulations, the LA-GA-T (look ahead genetic algorithm with taboo) proposed by Akdemir [2] in his STPGA (selection of training populations by genetic algorithms) R package. Genetic algorithms work based on the principles of biological evolution so that they solve their problems using evolutionary strategies, where at each iteration, the best individuals are selected to generate the next generation. Still, the term taboo indicates that the solutions recently tested will be avoided in the next attempts, avoiding unnecessary (repeated) evaluations, and limiting the number of iterations to reach convergence. Thus, LA-GA-T optimizes the selection, on a genetic basis, of the *n* genotypes informed by APY to compose our optimized training set (OTS) [18]. In this context, Mendonça and Fritsche-Neto [35] used the algorithm designed by Akdemir [2] to select the most representative genotypes to build a training population. Similar to our findings, they did not notice an increase in PA while using OTS but were able to reduce the budget.

Nevertheless, in the present work, we extended these algorithms to more complex relationship matrices, such as the Kronecker product (⊗) of the genomic relationship matrix (GRM), with the environmental variance and covariance matrix (W) and the traits of interest (T) (G ⊗ E ⊗ T or G ⊗ W ⊗ T). Even though these scenarios lead to a high level of imbalance in the data, they determine exactly what needs to be phenotyped to compose an optimized training set, which is called selective phenotyping [3]. In summary, it allowed us to identify which genotypes should be evaluated in which environments and for which traits to form a super-optimized training population, capable of predicting the performance of the entire population for all traits and environments, filling gaps in GS, and answering questions about the optimal partition of genotypes across environments [24].

Voss-Fels et al. [55] said that the TRS must be exceptionally large. Similarly, Wang et al. [56] stated that the larger the TRS, the better the estimation of genetic effects and, therefore, the greater the accuracy, mainly for low heritability traits. Here, the amount of information for each OTS, regardless of the kernel, is small, representing between 1.6 and 19.6% of the total available information. Moreover, the samples have an adequate distribution of genotypes, including those that perform well and those that are subpar, bringing positive impacts on PA [37]. As expected and similar to the findings of Pinho Morais et al. [43], PA is diminished with a small effective population size since the sample contains low genetic variability. However, we still obtained satisfactory prediction abilities, with an overall mean of 0.41 and 0.54 for USP and Helix, respectively. The advantage was that costs were reduced by more than 1000%, and the labor associated with phenotyping the TRS was also reduced. According to Krchov and Bernardo [26], accuracies should be greater than 0.50 so that the GS is superior and chosen instead of phenotypic selection.

It is interesting to notice that with similar increases in the sample size, of approximately 90-100% from OTS 1 to OTS 2, and from OTS 2 to OTS 3, the increases in PA were different for each kernel-dataset combination, as given in Figs. 3 and 4 and Table 7. PA practically doubled for GWT compared to GET, mainly for USP, where the amount of information used as TRS represents a small portion of the total dataset. Therefore, even a small increase in the training population can result in substantial PA increases, especially when considering the response to selection per dollar invested. Overall, GET proved less effective in optimizing TRS since it assumes that the environments are unrelated and require additional information to explain variations in the entire dataset. Meanwhile, GWT was more effective in optimizing TRS, creating a relationship matrix between environments via the W matrix. Furthermore, with all the aggregated information it carries, GWT can select individuals more assertively and consequently needs less information to form the OTS, which means that the size of TRS is smaller, therefore reducing costs and labor.

Considering that the cost of genotyping is decreasing, whereas the costs of field testing in maize are either stagnating or rising and knowing that genotypic information is stable and not influenced by seasonal variation, less effort is expended, saving resources, and making genotypic selection more efficient [26]. In addition, it is worth noting that with a fixed budget, as we decrease the training population size, through the use of OTS, we can have a larger test population, since the resources are reallocated from phenotyping to genotyping, or even, with a fixed training population, small budget increments mean a significant increase in the test population, which can be considerably translated to greater selection gains [26, 45].

Looking to reduce costs with phenotyping, Lado et al. [27] found that there was no loss in prediction ability when the training population was reduced to 30% when highly correlated traits were used in the multi-trait model since the correlation between the traits and the heritability of each contributed to the prediction of the others. Here, we were able to reduce the training population to approximately 4% of the original size, obtaining satisfactory results when considering the genetic gains per 10,000USD invested, which were about 0.70 × 10^− 3^ against 0.16 × 10^− 3^, with optimized (**GWT**) against standard scenario (MTMET CV2) TRS, respectively, for the HEL dataset and 1.42 × 10^− 3^ against 0.16 × 10^− 3^ for the USP dataset.

The genetic gain per dollar invested was estimated as a basis for comparing responses across different sizes of TRS datasets, and mainly to justify optimization to form training sets for GS. From the results, we can infer that the optimized populations have advantages over the standard scenario (70% TRS-30% TS). The difference in gains between the datasets is due to their specific characteristics, such as the number of inbred lines used as parents and the PA obtained. For the HEL dataset, although the PA was higher, the costs of genotyping were also higher, given the great number of inbred lines, resulting in lower gains. There was also an inversion of gains between GET and GWT, where the efficiency of the GWT kernel remains practically constant while that of GET drops. For the USP dataset, it was the opposite; the PA was lower, but there were fewer individuals for both genotype and phenotype. Thus, the costs were lower, and the gains were higher; also, GWT outperformed GET in all the scenarios. Altogether, GWT reduced the TRS by up to 58% compared with GET. From this point of view, although the PA using GWT is the lowest, regardless of the scenario, its advantage can be offset by the gain in response to selection per dollar invested (see Fig. 5)., giving special attention to GWT in the USP dataset. In summary, when comparing MTMET – CV2 with GWT, there was a reduction of up to 60% in terms of PA; however, it substantially reduces the number of plots and traits to be phenotyped by up to 98%. Furthermore, using OTS plus W (GWT) increases the response to selection per amount invested up to 142% compared with GET. Thus, there is no gain in PA with OTS, but the reduction in the training population greatly reduces costs and fieldwork, and consequently, the relative genetic gains are greater.

Comparing optimized populations against randomly selected individuals, when small training sets are used, as is the case of the OTS1 scenario, optimized training samples yielded greater PA than random samples of the same size, by 7.9% (Fig. 3) and 20.1% (Fig. 4), for HELIX and USP respectively. However, as the size of training sets increases, the differences between optimized and random samples are less pronounced. The same pattern was observed by Akdemir et al. [4], Akdemir [2] and Pinho Morais et al. [43].

Considering this, our results add to the subject of training sets, answering questions about which design to choose to distribute the population for evaluation and which individuals to choose to form the training population, because, as shown, TRS is key to the success of GS.

Multi-trait and multi-environment analyses have been applied to optimize the distribution of resources through GP, reaching satisfactory accuracy and gains. However, this scenario can still be improved, by taking advantage of new tools like the environmental relationship matrix (W matrix) and genetic algorithms (APY and LA-GA-T) to optimize resource allocation. To our knowledge, this is the first study to test the optimization of training set populations using genetic algorithms, which determine the size of the population and select the individuals based on complex kernels, causing a high level of imbalance. We observed that using a smaller optimized training set reduced the need for phenotypic evaluation in the field, saving money that can be reallocated for genotyping. Moreover, we estimated the genetic gains per 10,000USD invested, which indicated that, by applying optimization and maintaining a constant selection intensity, under a fixed budget, a greater number of breeding candidates could be tested per cycle and at early stages, increasing the gains.

The initial investments in GS are considerably high; however, they are offset by gains per unit of time. Nevertheless, it is known that the genetic variance of a given population decreases over the breeding cycles, especially in small population sizes, which limits the selection gains [41]. Hence, we can consider optimization aiming to renovate training sets each year to keep GS accuracy acceptable and raise the gains [21]. Therefore, periodic recalibration of the training population is important to endorse genetic variability, and when using an optimized population for recalibration, the cost of evaluation (genotyping plus phenotyping) is offset by the genetic gains obtained [41]. In summary, optimization yielded a satisfactory balance between gain versus costs and gain versus labor and provided new insight for using the algorithms tested here.

## Conclusions

Genomic prediction models that include G × E and G × W interaction effects always increase PA, performing better than main effects models; G × E interaction is the best scenario, with a small increase in multi-trait multi-environment analysis when compared with single-trait multi-environment analysis. Furthermore, our results indicate GWT kernel associated with genetic algorithms is efficient and can be proposed as a promising approach to designing optimized training sets for genomic prediction when the variance-covariance matrix of traits is available. Additionally, great improvements in the genetic gains per dollar invested were observed, suggesting that a good allocation of resources can be deployed. However, it is worth remembering that a specific interaction between combinations of germplasm, environments, experimental network, and evaluated traits must be considered when using the proposed approach.

## Methods

### Plant material

The phenotypic data consisted of two datasets of tropical maize single-cross hybrids. These datasets were chosen as a proof of concept and were extensively used and detailed by previous studies [5, 6]. Here we considered three traits: grain yield (GY, in ton ha^− 1^), plant height (PH, in cm), and ear height (EH, in cm). For GY assessment, ears were harvested at physiological maturity. Grains were weighed and adjusted to 13% moisture, and then the yield was corrected by area and plant population. PH and EH were measured from the ground to the flag leaf collar and from the ground to the base of the ear, respectively, on five representative plants within each plot. Below, we have a description of each one of the datasets.

### HEL dataset

The first dataset was composed of the phenotypic and genotypic data of 452 maize hybrids, obtained from single crosses in a partial diallel mating design among 106 tropical maize inbred lines, provided by Helix Seeds/Biomatrix (HEL), a seed company from the Agroceres group. A randomized complete block design (RCBD) with two blocks, two replications per genotype per location was used. Hybrids were evaluated over the 2014/15 growing season at three locations in Brazil: Ipiaçu (IP) (18° 41′ S, 49° 56′ W, 452 m above sea level), and Pato de Minas (PM) (18° 34′ S, 46° 31′ W, 832 m above sea level) in the state of Minas Gerais; and Sertanópolis (SE) (23° 03′ S, 51° 02′ W, 361 m above sea level) in the state of Paraná.

### USP dataset

The second dataset belongs to the University of Sao Paulo (USP). The data consisted of 903 maize single crosses, obtained from a diallel mating design between 49 inbred lines. Hybrids were evaluated at two locations in Brazil: Piracicaba (PI) and Anhumas (AN), in the state of São Paulo. They were evaluated for 2 years, during the second growing season of 2016 and 2017. The experimental design was an augmented randomized complete block design, with two commercial hybrids as checks per block. Although the locations are relatively close geographically, the soil and climate conditions are so contrasting that they characterize different environments, whereby each location × year combination was considered as an environment as follows: Anhumas ×2016 (AN.16), Piracicaba ×2016 (PI.16), Anhumas ×2017 (AN.17), and Piracicaba ×2017 (PI.17).

Given the sparse field trial design of both datasets, not all hybrids were evaluated at all locations. To balance the data, only hybrids evaluated at all locations for all traits were considered so that 247 and 623 genotypes remained for analysis, for HEL and USP, respectively. Balancing the data will later lead to the creation of controlled imbalances. Further details about both datasets can be found in [5, 6, 33].

### Genotypic data

Parental inbred lines from HEL and USP datasets were genotyped with an Affymetrix® Axiom® Maize Genotyping SNP array of 616 K [52]. The genomic quality control (QC) was performed using the SNPRelate R package [58]. Markers with a call rate ≤ 0.95 for HEL and a call rate ≤ 0.90 for USP, heterozygous loci in at least one of the parental lines, and monomorphic loci were removed.

The genotypic data of the hybrids were obtained by combining the homozygous markers of their parental lines. At first, allele frequencies and linkage disequilibrium were computed using the genotypes of the hybrids. Then, markers with minor allele frequency (MAF) ≤ 0.05 were removed. After QC, 30,467 and 62,409 high-quality SNPs were available to analyze the HEL and USP datasets, respectively. All the analyses were performed in the R software [44].

### Environmental data

ECs were obtained with the EnvRtype R package [13] and were used as descriptors of the environment to increase prediction ability in multi-environment GP scenarios. EnvRtype is a package to acquire and process weather data because it interplays the experimental network information, such as geographical coordinates (WGS84), and plant and harvest dates, the package collects and processes remote weather data from NASA Power databases. Also, this package enables the processing of environmental factors in actual covariates, which here were summarized according to the plant phenology intervals of growth or preestablished fixed-time intervals. We used five-time intervals according to the maize cycle phenology for this research, defined as 0-14, 15-35, 36-60, 61-90, and 91-120 days after emergence. The environmental factors used were radiation-related (sunshine hours, in hours; and total day length, in hours); radiation balance (insolation incident on a horizontal surface, shortwave and downward thermal infrared radiative flux, longwave); and atmospheric demands (rainfall precipitation, in mm; and relative air humidity, in %) as described in Costa-Neto et al. [12]. The ECs were estimated from mean air temperature and accumulated precipitation over the period and then used to establish G × E interaction. This process creates a covariate matrix of ECs called W, producing environmental relationship matrices for GP.

For the HEL dataset, each environment was characterized by 217 ECs. For the USP dataset, each environment was characterized by 238 ECs. The resulting matrices have dimensions of 3 × 217 and 4 × 238 for HEL and USP, respectively. They were then used to estimate the W matrix. In the enviromic kernel, the rows represent the environments, and the columns are the ECs.

Afterward, we can calculate an enviromic kernel, that is equivalent to a genomic relationship matrix but for environments, using the linear kernel approach proposed by Jarquín et al. [23]:

$${K}_E=\frac{WW^{\prime }}{tr\ \left(W{W}^{\prime}\right)/q}$$

where *K*_*E*_ is defined as the “enviromic-based kernel” accounting for the similarity between environments, *W* is the matrix of ECs, *tr* is the trace of the matrix and *q* is the number of environments [13]. *K*_*E*_ is *q* x *q* dimension.

### Scaling the traits

We established a transformation for EH that represents the distance from the actual EH to an ideal ideotype, defined here as 80 cm, according to the following formula:

$${EH}_{tr}=\left|{EH}_{ij}-80\right|\ast \left(-1\right)$$

where *EH*_*tr*_ is the transformed EH, and *EH*_*ij*_ is the EH for genotype *i* at environment *j*, in centimeters*.* According to this transformation, the closer to zero, the closer to our ideal height. The direction of selection for GY and EH is opposite, i.e., we want to select high-yielding varieties but with an ear at 80 cm, however, those traits are positively correlated. Therefore, the absolute value of the difference between the actual EH and the ideotype was multiplied by −1 to force a negative correlation between *EH*_*tr*_ and GY.

For PH, values were normalized to obtain a normal distribution interval. To fit the models, the phenotypic records were mean-centered and scaled to a normal distribution with *x*~*N*(0, 1) in which *x* is the trait (GY, PH, and EH).

### Statistical analysis

#### Phenotypic analysis

We used a linear mixed model for the two-step analysis to calculate the best linear unbiased estimates (BLUEs) of each trait’s hybrids. BLUEs were obtained across environments for the USP and HEL datasets by the following respective models:

$${\boldsymbol{y}}_{\boldsymbol{USPij}}=\boldsymbol{\mu} +{\boldsymbol{g}}_i+{\boldsymbol{g}}^{\ast }+\boldsymbol{bl}+{\boldsymbol{\varepsilon}}_{\boldsymbol{ij}}$$

$${\boldsymbol{y}}_{\boldsymbol{HELij}}=\boldsymbol{\mu} +{\boldsymbol{g}}_i+\boldsymbol{bl}+{\boldsymbol{\varepsilon}}_{\boldsymbol{ij}}$$

where ***y***_***ij***_ is the estimated phenotypic value of the *i*^*th*^ genotype at the *j*^*th*^ environment, μ is the general mean or intercept, ***g***_***i***_ is the fixed effect of the hybrid genotype *i*, g^*^ is the fixed effect of check genotypes, ***bl*** is the random effect of blocks for the USP dataset (bl ~ NM (0, ${\upsigma}_{bl}^2$) and the fixed effect of blocks for the HEL dataset, and finally, ε_***ij***_ is the residual error for genotype *i* at environment *j*, where 𝜀 ~𝑁M(0,𝜎^2^).

The blocking effect was considered as random for USP dataset given the nature of the augmented blocks experimental design, while it was considered as fixed for HEL dataset because the RCBD contained only two blocks, which is not a representative sample size to be considered as random.

Phenotypic analyses were performed using the ASReml-R package [8] of R software [44] and subsequently used in our genomic prediction models.

The variance components estimated for each model’s effect were used to estimate the average broad-sense heritability (H^2^).

### Genomic prediction scenarios

We tested three different genomic prediction scenarios: single-trait multi-environment trials (STMET) and multi-trait multi-environment trials (MTMET), with 70% of data used as TRS; and optimized training sets (OTS), with varying percentages of data used as TRS. One of our objectives was to obtain PA for OTS. However, to make inferences about economics and the advantages of using the proposed approach, we needed reference values for comparison, hereafter referred to as benchmarks.

To obtain the benchmark values of PA for the models, we tested them (described in section 2.5.3) in full STMET and MTMET genomic prediction analyses through cross-validation schemes with replication (described in section 2.6). From those results, we obtained the highest possible PA for the specific datasets used in this study, using all the phenotypic information available [18].

For the OTS, two different kernels were built from the Kronecker product (⨂) between the variance-covariance matrices (Σ) of genotypes (G), environments (E), environmental covariates matrix (W), and traits (T) as follows: Σ_G_ ⨂ Σ_E_ ⨂ Σ_T_ and Σ_G_ ⨂ Σ_W_ ⨂ Σ_T_, hereafter called GET and GWT, respectively. These kernels, used as inputs for the algorithms, assemble combinations between our variables.

For comparison matters, random samples with the same size of the OTS for the different kernels were also used as training sets in the prediction model. After sampling, a hybrid was added as a check. According to sample sizes, Random1 corresponds to OTS1, Random2 to OTS2, and Random3 to OTS3, described subsequently.

### The genetic algorithms for training set optimization

The algorithms APY [38, 39] and LA-GA-T, from the STPGA R package [2] were used in optimization scenarios. First, APY determines samples’ size by singular value decomposition, giving the number of components that explain 98% of the variation within the population. Next, LA-GA-T, a genetic-based algorithm, selects representative individuals from the population to compose the samples. Thus, from the population size established by APY, LA-GA-T sample individuals with representative information within the respective datasets. Once we had the sample size obtained from APY, three samples with replacement were randomly taken from LA-GA-T, to minimize the stochastic error and increase the reliability.

**Fig. 6:**
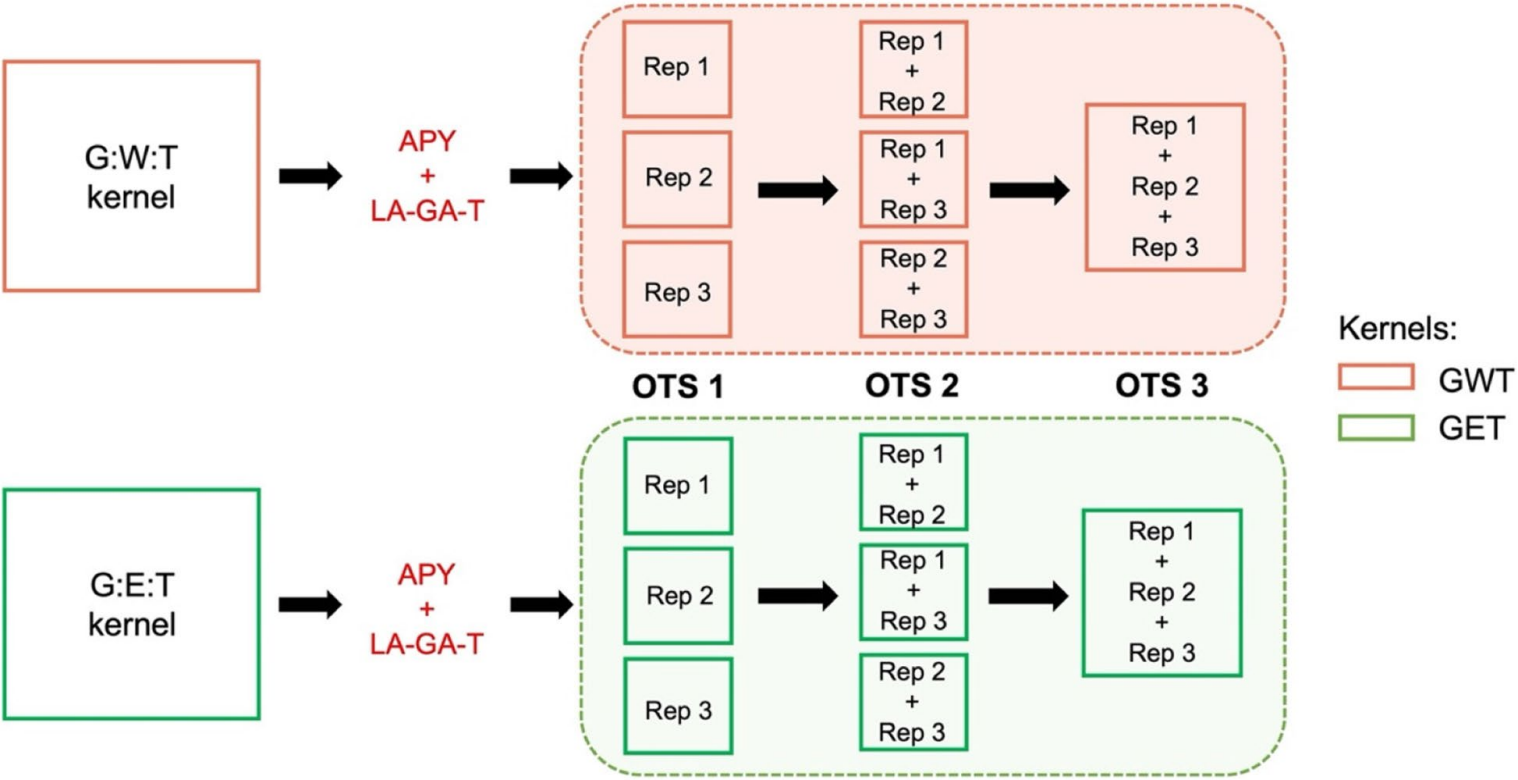
Scheme illustrating how the optimized training set populations were formed. The algorithms APY (to define the number of information) and LA-GA-T (to select information optimally) were applied to the kernels GWT (genotype *x* environmental covariates *x* trait) and GET (genotype *x* environment *x* trait) to obtain three replications for each kernel, which formed our OTS 1 (optimized training set 1). From OTS 1, two other scenarios were created: OTS 2 and OTS 3. The whole process was done for the two datasets, HEL and USP

These three samples were taken for each kernel (GET and GWT) × dataset (HEL and USP), and constitute our optimized training set 1, hereafter referred to as OTS 1. At each model iteration, one of the three samples was used as TRS, and the remaining information was used as TS. In addition, two other scenarios were created from the original samples. The first one was created by combining two samples at a time, resulting the scenario OTS 2. In the second one, the three samples of OTS 1 were combined, creating OTS 3, resulting in just one bigger TRS, when compared to OTS 1 and OTS 2. (see Fig. 6).

It is important to clarify that, for training set optimization, we used the two kernels, GET and GWT, to obtain specific combinations of genotype × environment × trait that compose our OTS. For that reason, sampled individuals were trait and environment-specific, and dependent on the kernel. Moreover, a hybrid was added as a check, and therefore its full phenotypic information (i.e., all environments × traits combinations) was included to create connectivity between environments.

### Genomic prediction via single and multi-trait multi-environment models with additivity and dominance effects

The genomic prediction was first performed by five GBLUP additive + dominance models for STMET and MTMET scenarios. The following models were previously described and tested. For further details, see Costa-Neto et al. [14].

### Model 1 (M1): environment and main additive plus dominance genomic effects (EAD)

M1 is the most basic model tested, and is described as follows:

$${\boldsymbol{y}}_{\boldsymbol{t}}={\boldsymbol{Z}}_E\beta +{\boldsymbol{Z}}_G{\boldsymbol{u}}_{\boldsymbol{A}}+{\boldsymbol{Z}}_G{\boldsymbol{u}}_{\boldsymbol{D}}+\boldsymbol{\varepsilon}$$

where ***y***_***t***_ is the adjusted observed values (BLUEs) correspondent to the ***t*** trait, obtained from the first step for the hybrids. The fixed effects of the environment were modeled by ***Z***_***E***﻿_ ﻿ *β*, with the incidence matrix ***Z***_***E***_***,*** and ***Z***_*G*_ is the incidence matrix for the genotypic effects. ﻿***u***_***A***_ is the vector of additive genetic effects, where ﻿***u***_***A***_ ~***MN*** (**0,*G***_***a***_${\sigma}_A^2$), ﻿***u***_***D***_ is the vector of dominance effects, where ﻿***u***_***D***_ ~***MN*** (**0,*G***_***d***_${\sigma}_D^2$), and ***ε*** is the random residual effect, where ***ε*** ~***MN*** (**0,**${\sigma}_e^2\boldsymbol{I}\Big)$. ***G***_***a***_ and ***G***_***d***_ are the genomic relationship matrices (GRM) for additive and dominant effects, respectively, given as follows:

$${\boldsymbol{G}}_{\boldsymbol{a}}=\frac{W_A{W}_A^{\prime }}{2{\sum}_i^n{p}_i\left(1-{p}_i\right)}$$

where the values from the incidence matrix ***W***_***A***_ are equal to *0, 1* and *2*, for genotypes markers of *A*_*1*_*A*_*1*_*, A*_*1*_*A*_*2*_ and *A*_*2*_*A*_*2*_, respectively, and *p*_*i*_ is the frequency of one allele from the *i*^*th*^ locus [53].

$${\boldsymbol{G}}_{\boldsymbol{d}}=\frac{W_D{W}_D^{\prime }}{4{\sum}_i^n{\left\{\left({p}_i\left(1-{p}_i\right)\right.\right\}}^2}$$

where ***W***_***D***_ contains values equal to 0 − 2q^2^, 2pq, and 0 − 2p^2^ for *A*_*1*_*A*_*1*_, *A*_*1*_*A*_*2*_ and *A*_*2*_*A*_*2*,_ respectively [54]*.*

### Model 2 (M2): environment, main effects + block diagonal GE (EAD + GE)

This model is an update of M1 that accounts for the main effects (A and D), adding the additive × environment and dominance × environment interactions effects (AE and DE).

$${\boldsymbol{y}}_{\boldsymbol{t}}={\boldsymbol{Z}}_{\boldsymbol{E}}\beta +{\boldsymbol{Z}}_G{\boldsymbol{u}}_{\boldsymbol{A}}+{\boldsymbol{Z}}_G{\boldsymbol{u}}_{\boldsymbol{D}}+{\boldsymbol{u}}_{\boldsymbol{A}\boldsymbol{E}}+{\boldsymbol{u}}_{\boldsymbol{D}\boldsymbol{E}}+\boldsymbol{\varepsilon}$$

where ***u***_***AE***_ and ***u***_***DE***_ are the vectors of random effects of the interactions. ***u***_***AE***_ and ***u***_***DE***_ have a multivariate normal distribution, ***u***_***AE***_ ~ ***MN*** (**0,[**${\boldsymbol{Z}}_G{\boldsymbol{G}}_{\boldsymbol{a}}{{\boldsymbol{Z}}_G}^{\prime}\left]\odot \left[{\boldsymbol{Z}}_{\boldsymbol{E}}{{\boldsymbol{Z}}_{\boldsymbol{E}}}^{\prime}\right]\ {\sigma}_{ae}^2\right)$ and ***u***_***DE***_ ~ ***MN*** (**0,[**${\boldsymbol{Z}}_{\boldsymbol{G}}{\boldsymbol{G}}_{\boldsymbol{d}}{{\boldsymbol{Z}}_G}^{\prime}\left]\odot \left[{\boldsymbol{Z}}_{\boldsymbol{E}}{{\boldsymbol{Z}}_{\boldsymbol{E}}}^{\prime}\right]\ {\sigma}_{de}^2\right)$, where ${\boldsymbol{\sigma}}_{\boldsymbol{ae}}^{\textbf{2}}$ and ${\boldsymbol{\sigma}}_{\boldsymbol{de}}^{\textbf{2}}$ are the variance components for ***u***_***AE***_ and ***u***_***DE***_ interaction effects, respectively, and ⊙ is the Hadamard product [6, 23, 29].

### Model 3 (M3): Main effects + main environmental covariable information (EAD + W)

This third model includes environmental covariable information (W) from envirotyping data.

$${\boldsymbol{y}}_{\boldsymbol{t}}={\boldsymbol{Z}}_{\boldsymbol{E}}\beta +{\boldsymbol{Z}}_G{\boldsymbol{u}}_{\boldsymbol{A}}+{\boldsymbol{Z}}_G{\boldsymbol{u}}_{\boldsymbol{D}}+{\boldsymbol{u}}_{\boldsymbol{W}}+\boldsymbol{\varepsilon}$$

where ***u***_***W***_ is the matrix of environmental covariates. According to Costa-Neto et al. [14], it is a piece of non-genetic information that helps to explain phenotypic variation across environments.

### Model 4 (M4): Main effects EADW + reaction norm for GE (EAD + W + GE)

This model extends the previous model (M3), adding the environment’s additive and dominance interactions.

$${\boldsymbol{y}}_{\boldsymbol{t}}={\boldsymbol{Z}}_{\boldsymbol{E}}\beta +{\boldsymbol{Z}}_G{\boldsymbol{u}}_{\boldsymbol{A}}+{\boldsymbol{Z}}_G{\boldsymbol{u}}_{\boldsymbol{D}}+{\boldsymbol{u}}_{\boldsymbol{W}}+{\boldsymbol{u}}_{\boldsymbol{A}\boldsymbol{E}}+{\boldsymbol{u}}_{\boldsymbol{D}\boldsymbol{E}}+\boldsymbol{\varepsilon}$$

### Model 5 (M5): Main effects EAD + W + reaction norm for GW (EAD + W + GW)

This model modifies the latter (M4) reaction-norm variation; it replaces the genomic × environment interactions with the genomic × enviromic effects interactions.

$${\boldsymbol{y}}_{\boldsymbol{t}}={\boldsymbol{Z}}_{\boldsymbol{E}}\beta +{\boldsymbol{Z}}_G{\boldsymbol{u}}_{\boldsymbol{A}}+{\boldsymbol{Z}}_G{\boldsymbol{u}}_{\boldsymbol{D}}+{\boldsymbol{u}}_{\boldsymbol{W}}+{\boldsymbol{u}}_{\boldsymbol{A}\boldsymbol{W}}+{\boldsymbol{u}}_{\boldsymbol{D}\boldsymbol{W}}+\boldsymbol{\varepsilon}$$

where ***u***_***AW***_ and ***u***_***DW***_ are the vectors of random effects of interactions. ***u***_***AW***_ and ***u***_***DW***_ have a multivariate normal distribution, ***u***_***AW***_ ~ ***MN*** (**0, [*Z***_*G*_***G***_***a***_${{\boldsymbol{Z}}_G}^{\prime}\left]\odot \left[{\boldsymbol{WW}}^{\prime}\right]\ {\sigma}_{aw}^2\right)$ and ***u***_***DW***_ ~ ***MN*** (**0, [*Z***_*G*_*G*_*d*_${{\boldsymbol{Z}}_G}^{\prime}\left]\odot \left[{\boldsymbol{WW}}^{\prime}\right]\ {\sigma}_{dw}^2\right).$ Here we can assume that there are different levels of relationship between genotypes and environments.

All models were fitted with the Bayesian Generalized Linear Regression BGLR R package [42], using a Gibbs sampler with 10,000 iterations, assuming a burn-in of 1000, and a thinning of 2.

It is important to point out that the “Multitrait” function of the BGLR package has some basic premises that must be met for the model to work, one of them is the availability of complete information from at least one genotype. Here, as we used multiple traits with the multiple environments approach, we selected a genotype as a check, with complete phenotypic data available, common to all environments. Additionally, the covariance matrix between traits was assumed as known, and therefore the covariance matrices between traits of the full datasets were predefined and used for the multi-trait multi-environment under OTS and Random prediction scenarios.

### Assessing the prediction ability of the models

The PA of GP models for STMET and MTMET was assessed using two cross-validations schemes (CV), proposed by Burgueño et al. [7]. The first cross-validation scheme, CV-1, considered 50 random partitions where 70% of the hybrids were used as TRS, with genotypic and phenotypic information available (hybrids phenotyped for all traits in all environments), while the remaining 30% (non-phenotyped hybrids) were used as TS, with only genotypic information available. CV-1 aims to quantify the PA of GP models by reproducing a scenario frequently faced by breeders when predicting new genotypes in a network of known environments, i.e., newly developed maize hybrids never evaluated. The second cross-validation scheme, CV-2, mimics another common situation where genotypes are tested in unbalanced field trials (or incomplete field trials), i.e., some genotypes are evaluated in some environments but not in the entire experimental network. For CV-2, we also considered 50 random partitions with 70% of the data as TRS and the remaining 30% as TS.

We assessed PA in two ways: 1) per trait only, where the PA is the average between environments for each trait, and 2) per trait per environment. In this context, PA is the average Pearson’s correlation coefficient of all TRS-TS partitions, calculated between the genomic estimated breeding values (GEBVs) and the estimated genetic values (BLUEs) of TS individuals within trait or within trait and environment. Finally, PAs were used to compare the performance of each model.

For OTS and Random scenarios, we did not use cross-validation schemes. The samples used as TRS already gave us a high imbalance of information, sometimes mimicking CV1, where the genotype was not observed in any environment, and sometimes CV2, where the genotype was evaluated in some environments but not in others. PA was calculated as the Pearson’s correlation coefficient between the predicted value and the BLUE of the TS individuals within each environment for each trait; then the average across traits was calculated, like in the previous scenarios.

### Response to selection per unit of dollar invested

We finally estimated the response to selection per unit of dollar invested by comparing the efficiency of the different scenarios tested in this study with pure phenotypic selection (PS). The methodology was based on Krchov & Bernardo [26] and Muleta et al. [41]. Phenotyping costs per plot were assumed to be 2 US dollars (USD) for PH and EH, and 4 USD for GY. For genotyping, we considered 20 USD per sample. Due to the F1 nature of the hybrids, we only genotyped the parental inbred lines and assembled the hybrid genotype in silico. Therefore, the total cost consisted of genotyping costs (20 USD per line) plus the cost of phenotyping the TRS, which varied across scenarios. This calculation was done for each dataset × OTS scenario (OTS 1, OTS 2, and OTS 3) × kernel (GET vs GWT), and for benchmarks (dataset × MTMET-CV2). For PS, we assumed a prediction ability equal to 1 for all traits. The accuracy (PA/ $\sqrt{H^2}$, being *H*^2^ trait specific) was then calculated separately for each trait. The genetic gain was then estimated by dividing the average accuracy obtained for each scenario (under M4) by the corresponding total cost, genotyping + phenotyping for GP scenarios, and only phenotyping cost for PS; given that all other components of the breeder’s equation [32] were considered as fixed. Response to selection per unit of dollar invested was subsequently transformed to a base of 10,000 USD.

## Data Availability

The datasets supporting the conclusions of this article are available in the Mendeley Data repository, https://data.mendeley.com/datasets/hj73jcgw97/2
